# Safety and Efficacy of Fecal Microbiota Transplant in Chronic Pouchitis—A Systematic Review With Meta-Analysis

**DOI:** 10.1016/j.gastha.2023.04.005

**Published:** 2023-04-25

**Authors:** Tamara Kahan, Saurabh Chandan, Shahab R. Khan, Smit Deliwala, Shannon Chang, Jordan Axelrad, Aasma Shaukat

**Affiliations:** 1 Division of Gastroenterology and Hepatology, NYU Grossman School of Medicine, New York, New York; 2 Division of Gastroenterology and Hepatology, Creighton University School of Medicine, Omaha, Nebraska; 3 Department of Medicine, Brigham and Women’s Hospital, Harvard Medical School, Boston, Massachusetts; 4 Division of Gastroenterology & Hepatology, Emory University, Atlanta, Georgia

**Keywords:** Pouchitis, FMT, Fecal Microbiota Transplant

## Abstract

**Background and Aims:**

Pouchitis is the most common long-term complication after ileal-pouch anal anastomosis in patients with ulcerative colitis. We conducted a systematic review and meta-analysis evaluating the safety and efficacy of fecal microbiota transplant (FMT) in chronic antibiotic dependent and refractory pouchitis.

**Methods:**

Multiple databases were searched through April 2022 for studies that reported the efficacy and safety of FMT in patients with chronic pouchitis. Meta-analysis using random effects model was performed to calculate pooled rates.

**Results:**

Eight studies with a total of 89 patients were included in our review, with 74 patients having received FMT and 15 patients having received placebo. The mean age ranged from 32.6 to 51.5 years. In patients that received FMT, the pooled rates of overall remission was (Pouchitis Disease Activity Index score < 7) 22% (95% CI, 9%–43%; I^2^, 29%), clinical remission was 20% (95% CI, 6%–49%; I^2^, 25%), clinical response rate was 42% (95% CI, 30%–54%; I^2^, 7%), and the relapse rate 60% (95% CI, 40%–77%, I^2^ 16%) over the mean follow up of 4.67 months (range 1–12 months). The pooled proportion of patients with adverse events was 54% (95% CI, 21%–84%; I^2^, 73%). There were no serious adverse events or deaths.

**Conclusion:**

In patients with chronic pouchitis, FMT is safe though there are mixed results in terms of its long-term efficacy. Future Randomized Controlled Trials with larger sample sizes and greater standardization in terms of preparation, delivery, and length of treatment of FMT are needed to determine efficacy.

## Introduction

Inflammatory bowel diseases, including Crohn’s disease and ulcerative colitis (UC), are chronic inflammatory conditions of the gastrointestinal tract characterized by dysregulated immune responses to an altered gut microbiome in genetically susceptible individuals. Despite the recent emergence of more effective and durable medical therapies for UC, a significant proportion of patients will require colectomy during their disease course. Surgical interventions for medically refractory UC, particularly proctocolectomy with ileal pouch anal anastomosis, are often complicated by pouchitis, an idiopathic inflammatory condition of the ileal pouch.^1^^,^^2^ Pouchitis, which has an estimated lifetime prevalence of 70%, is characterized by sudden onset of increased stool frequency, urgency, incontinence, abdominal pain, and malaise.^3^ While the pathogenesis of pouchitis is poorly understood, surgical alterations of the bowel anatomy are thought to affect the microbiome of the terminal ileum.^2^ Specifically, the microbiome of an inflamed pouch is characterized by decreased bacterial diversity and changes in the abundance of several bacterial species.^4^ Further evidence for the involvement of the gut microbiome comes from successful treatment of acute pouchitis with antibiotics (typically ciprofloxacin and/or metronidazole) and secondary prophylaxis through probiotic use.^3^^,^^5^ However, the 10%–15% of pouchitis cases that become chronic are significantly more difficult to manage and often require long-term antibiotics and/or immunosuppressive therapies such as biologics or small molecule therapies.^6^ At present, there is limited treatment for chronic antibiotic-dependent or antibiotic-refractory pouchitis. While biologic therapies and small molecule therapies have been tried with variable rates of success,^7^ pouch failure may occur which requires surgical redo or excision of the ileal pouch, with 11% of pouch excisions linked to chronic pouchitis.^8^^,^^9^

Due to its success in treating refractory and recurrent *Clostridioides difficile* infection, fecal microbiota transplantation (FMT), where a fecal suspension from a healthy individual is infused into the gastrointestinal tract of a patient with pouchitis, has been explored as a potential treatment.^10^ Given the link between the gut microbiome and pouchitis, FMT is of particular interest due to its potential to restore a normal intestinal microbial environment. However, studies exploring the use of FMT in treating pouchitis have yielded mixed results. Adverse effects to FMT are often short-term, and include transitional bowel discomfort, fever, and abdominal pain.^11^ Finding an alternative treatment to chronic antibiotic or immunosuppressive therapy in pouchitis, such as FMT, may improve patients’ quality of life, reduce antibiotic resistance, need for long term therapies, and adverse effects, and reduce further complications affecting the pouch. The following systematic review and meta-analysis assesses the current literature on treatment of chronic pouchitis with FMT, focusing on clinical outcomes and adverse effects.

## Methods

### Search Strategy

The literature was searched by 2 individuals, medical librarian (C.P.) and author (T.K.), for studies that reported the use of FMT as a treatment for pouchitis. A comprehensive search of several databases from inception to April 2022 was performed. The databases included Ovid Embase (1974+), Ovid Medline (1946+ including epub ahead of print, in-process & other non-indexed citations), PubMed, and Cochrane. Manual search for studies of interest was performed by 2 authors (T.K., S.C.). Controlled vocabulary supplemented with keywords was used to search for studies of interest. The search strategies were created using a combination of keywords and standardized index terms. Keywords included “fecal microbiota transplant,” “FMT,” “pouchitis,” and “proctocolectomy.” Results were limited to English language. All results were exported to EndNote (Clarivate) where 272 obvious duplicates were removed leaving 1664 citations. Details of the search are provided in Supplementary Appendix-1. Details of study selection are provided in the Preferred Reporting Items for Systematic Reviews and Meta-Analyses Flow Chart—Figure A1. The full search strategy is available in Supplementary Appendix-1. The Meta-analysis of Observational Studies in Epidemiology and Preferred Reporting Items for Systematic Reviews and Meta-Analyses checklists were followed and are provided as Supplementary Appendix-2 and 3.^12^^,^^13^ The references of evaluated studies were examined to identify other studies of interest. The research protocol and patient, intervention, comparator and outcome framework were created prior to conducting a formal search strategy. We did not formally register our research protocol.

### Study Selection

After all search results were exported to EndNote, the titles and abstracts of potentially relevant articles were reviewed independently by 2 authors (T.K. and S.C.). Studies were categorized as either eligible or ineligible for inclusion, described in more detail below. The full texts of articles that met inclusion criteria were independently reviewed in full by 2 authors (T.K. and S.C.). There were no conflicts that needed to be a resolved by a third author. In this meta-analysis, we only included studies that reported on efficacy of treatment of FMT in patients with chronic pouchitis. Studies were included irrespective of inpatient/outpatient setting, study sample-size or design, follow-up time, and geography as long as they provided the clinical outcomes data needed for the analysis.

Our exclusion criteria were as follows: (1) studies that did not report on the clinical outcomes of interest, (2) individual case reports, (3) studies performed in the pediatric population (Age < 18 years), (4) studies examining Crohn’s-like disease of the pouch. We aimed at assessing the following with our analysis-

1. In adult patients with chronic pouchitis, how effective is FMT, compared to no treatment, in achieving response and remission, as assessed by improvement in Pouchitis Disease Activity Index (PDAI) score or its subscores?
2. In adult patients with chronic pouchitis, how effective is FMT, compared to antibiotics, in achieving response and remission, as assessed by improvement in PDAI score or its subscores?
3. In adult patients with chronic pouchitis, how effective is FMT, compared to probiotics, in achieving response and remission, as assessed by improvement in PDAI score or its subscores?
4. In adult patients with chronic pouchitis, how effective is FMT after antibiotics, compared to antibiotics alone, in achieving response and remission, as assessed by improvement in PDAI score?
5. In adult patients with chronic pouchitis, how effective is FMT, compared to biologics and small molecule therapies, in achieving response and remission, as assessed by improvement in PDAI score or its subscores?
6. How effective is FMT, compared to no treatment, at preventing recurrent pouchitis in adult patients as assessed by improvement in PDAI score or its subscores?
7. What are the associated adverse effects up to 6 months following FMT in adult patients with chronic pouchitis?

### Data Abstraction and Quality Assessment

Data on study-related outcomes from the individual studies were abstracted independently onto a standardized form by 2 authors (S.C., T.K.). Authors (S.C., T.K., S.R.K.) cross-verified the collected data for possible errors and 2 authors (S.C., S.R.K.) did the quality scoring independently. The National Institute of Health Quality Assessment Tool was used to assess the quality of cohort studies and case series, Table A1A and B.^14^ The quality of evidence presented in the Randomized Controlled Trials (RCTs) and risk of bias in all included studies was assessed using the Cochrane Risk of Bias Assessment Tool presented as Figure A2.^15^

### Outcomes Assessed and Definitions

We assessed studies of patients with chronic pouchitis (including the subtypes of chronic antibiotic-refractory pouchitis and chronic antibiotic-dependent pouchitis). We assessed clinical improvement and remission for all patients at the maximum follow up period. There was some variability in the definition of each outcome as follows:

A. Overall Remission—Defined as total PDAI score <7 at maximum follow up.
B. Clinical Response—Defined as subjective improvement in patient symptoms and/or reduction in PDAI ≥3 at first follow up.
C. Clinical Remission—Defined as modified PDAI <4 and no need for antibiotics, sustained improvement in clinical PDAI or subjective improvement in symptoms at maximum follow up.
D. Relapse—Recurrence of symptoms after last FMT administration.
E. Overall Adverse Events—As individually defined and reported by studies.

### Statistical Analysis

We used meta-analysis techniques to calculate the pooled estimates and 95% confidence intervals (CIs) in each case following the methods suggested by DerSimonian and Laird using the random-effects model.^16^ When the incidence of an outcome was zero in a study, a continuity correction of 0.5 was added to the number of incident cases before statistical analysis.^17^ Heterogeneity between studies was assessed by means of a *χ*^2^ test (Cochran Q statistic) and quantified with the I^2^ statistics. In this, values of <30%, 30%–60%, 61%–75%, and >75% were suggestive of low, moderate, substantial, and considerable heterogeneity, respectively. Publication bias, if warranted, was ascertained, qualitatively, by visual inspection of funnel plot and quantitatively, by the Egger test.^18^ When publication bias was present, further statistics using the fail-Safe N test and Duval and Tweedie’s ‘Trim and Fill’ test was used to ascertain the impact of the bias.^19^ A *P* value of <.05 was used ‘a priori’ to define significance between the groups compared.

All analyses were performed using Comprehensive Meta-Analysis (CMA) software, version 3 (BioStat, Englewood, NJ) and R-software version 4.1.3 (metafor package).

## Results

### Search Results and Population Characteristics

From an initial pool of 1935 studies, 1450 records were screened after duplicates were removed. Eight studies with a total of 89 patients were included in our review, with 74 patients having received FMT and 15 patients having received placebo. There were a total of 35 males and 40 females in our analysis, with the gender of 14 patients not documented. The mean age ranged from 32.6 to 51.5 years. The study follow-up time ranged from 1 month to 12 months (mean: 4.67 months).

**Figure 1:**
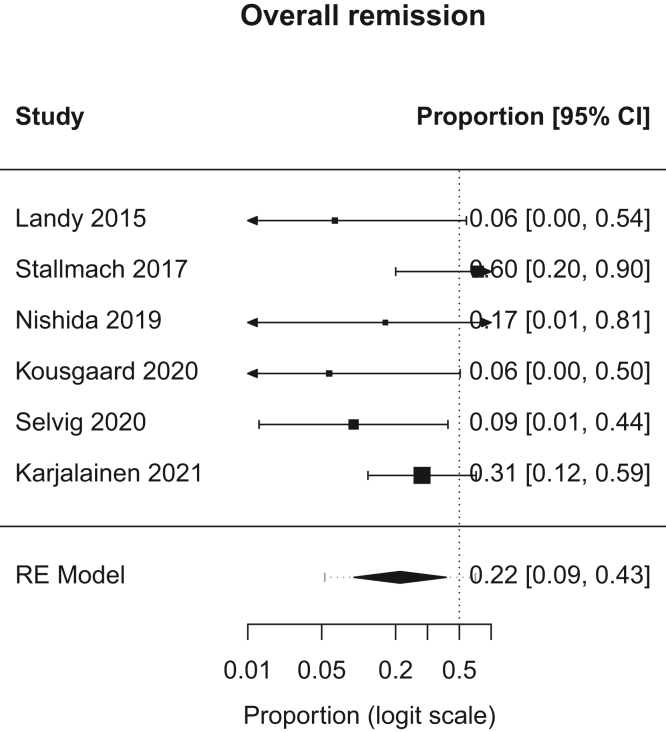
Forest plot, overall remission. RE, random effects.

**Figure 2:**
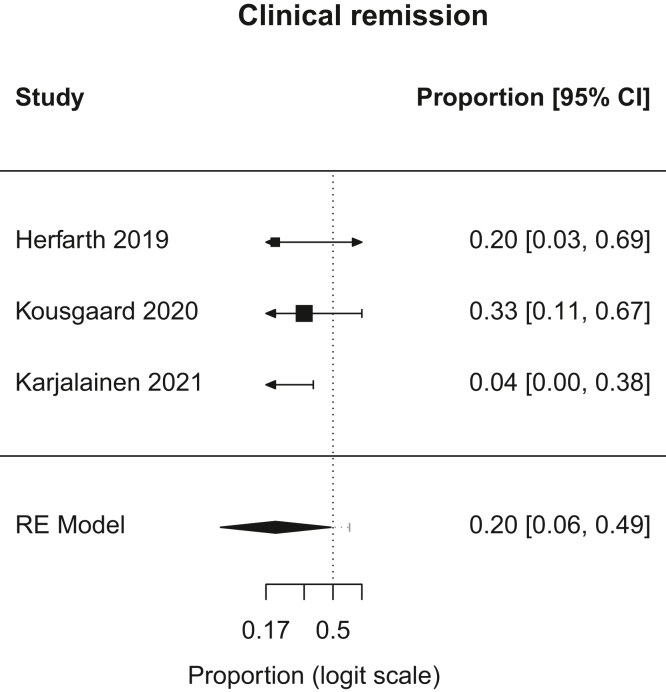
Forest plot, clinical remission. RE, random effects.

**Figure 3:**
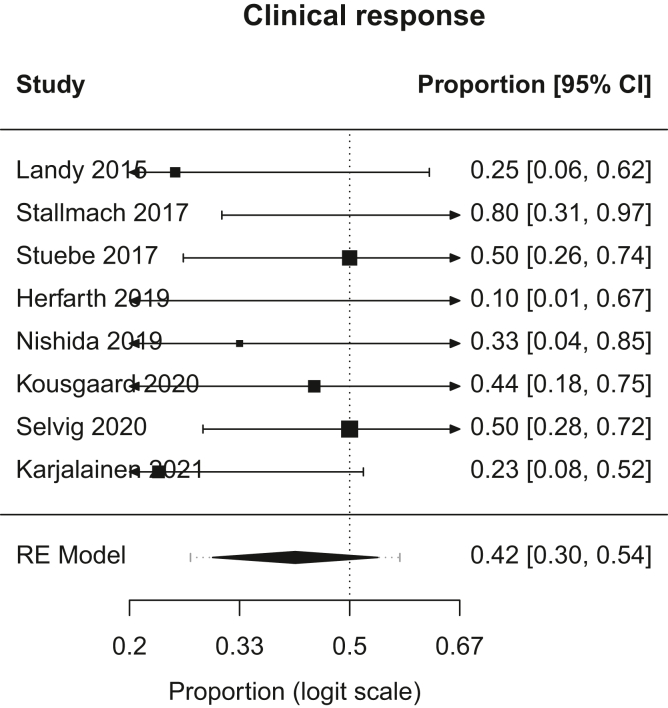
Forest plot, clinical response. RE, random effects.

**Figure 4:**
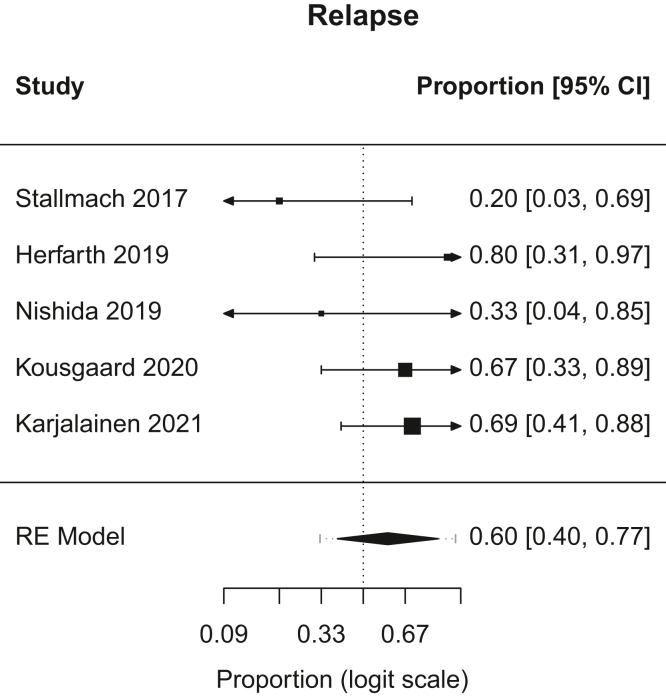
Forest plot, relapse. RE, random effects.

FMT route of administration varied across studies, with 2 studies using upper endoscopy, 1 study using nasogastric tube, 1 study using pouchoscopy, 1 study using colonoscopy, and 1 study administering enema. Two studies involving repeated dosing of FMT—1 through endoscopy followed by daily oral FMT for 2 weeks and 1 through endoscopy followed by transanal catheter administration of FMT 4 weeks later. Dosage of FMT ranged from 18 g of fecal material to 200 g of fecal material, with mean dosage 62 g of fecal material. Antibiotic use prior to FMT varied across the studies, with patients in some studies stopping antibiotics at least 24 hours prior to FMT^20^ while patients in other studies abstaining from antibiotic use for 2 weeks prior to FMT,^21^ and antibiotic use prior to FMT not documented in other studies.

### Characteristics and Quality of Included Studies

Of the studies included, 6 were prospective studies (including 2 RCTs), 3 were case series, and 1 was reported as a conference abstract only. Two studies originated from the United States while the others were from Europe. Based on NIH quality assessment tool, all case series were considered to be good quality. While two cohort studies were considered of good quality, Stuebe et al.^22^ was rated as poor quality. Based on Grading of Recommendations Assessment, Development and Evaluation methodology, overall grade of evidence as reported by the RCTs was high. The studies examined patients with chronic antibiotic-refractory pouchitis, chronic antibiotic-dependent pouchitis, or chronic pouchitis (unspecified). Further details of the studies are presented in Tables 1 and 2.

**Table 1:** Study Details and Patient Characteristics

Study	Design	Etiology	Intervention	Total patients (N)	Age (y, mean)	Sex (M/F)	Pre-FMT abx therapy	FMT route	Dosage
Landy 2015^21^	Prospective, NR, single center, UK	Chronic pouchitis	FMT	8	46 (24–63 range) [median]	3/5	No abx 2 wk prior to FMT	NG administration	30 g of stool was homogenized with 50 mL of 0.9% saline to produce a faecal-saline solution and 30 mL of the faecal-saline solution was administered via the nasogastric tube
Stallmach 2016^23^	Case series, NR, single center, Germany	Chronic antibiotic refractory pouchitis	FMT	5	32.60 (26–40)	2/3	All patients received abx, but unclear time course prior to FMT	Upper endoscopy	150 g of stool was homogenized with 400 mL of isotonic sodium chloride and half of this “slurry” was administered
Stuebe 2017^22^	Prospective, NR, single center, Germany	Chronic antibiotic refractory pouchitis	FMT	14	NR	NR	Not documented	Encapsulated cryopreserved microbiota or via endoscopic jejunal application	--
Herfarth 2019^20^	Prospective, RCT, single center, USA	Antibiotic dependent pouchitis	FMT vs placebo	6	37.33 (22–60)	4/2	Abx stopped at least 24 h before FMT	Single endoscopic FMT followed by daily oral FMT	2 x 30 mL, total of 24 g of donor stool followed by daily active FMT (6 G3 capsules gave a total of 4.2 g of donor stool)
Nishida 2019^24^	Case series, January 2015–June 2016, single center, Japan	Chronic pouchitis	FMT	3	40.33 (24–52)	2/1	All patients received abx, but unclear time course prior to FMT	Colonoscopy	150–200 g feces from donors was dissolved in 500 mL of sterile physiological saline (350–500 mL)
Kousgaard 2020^8^	Prospective, May 2018 to October 2018, single center, Denmark	Chronic pouchitis	FMT	9	51.5	3/6	All patients received abx, but unclear time course prior to FMT	Enema	100 mL suspended fecal material daily × 14 d
Selvig 2020^2^	Prospective, May 2015–January 2018, single center, USA	Chronic pouchitis	FMT	18	45 (34.25–56.25 IQR) [median]	6/12	7 patients received 5 d rifaximin to facilitate engraftment, 4 patients had new courses of antibiotics within 4 wk of FMT	Pouchoscopy	250 cc of donor fecal suspension (25 g of stool)
Karjalainen 2021^25^	Prospective, RCT, December 2017–August 2018, single center, Finland	Chronic pouchitis	FMT vs placebo	26	42.7 (10.2)	15/11	Abx stopped at least 36 h before FMT	First through flexible endoscopy, second through transanal catheter	170 mL of prepared fecal transplant containing 30 g of fecal material

Abx, antibiotics; NR, not reported.

**Table 2:** Study Details—Outcomes and Adverse Events

Study	Overall remission (PDAI < 7)	Relapse	Outcomes			PDAI score		Adverse events	Infection post-FMT	Follow-up (m: months)
			Response	Clinical remission	Endoscopic response	Pre-FMT	Post-FMT	FMT		
Landy 2015^21^	0/8 (total PDAI <7 at 4 wk)	--	2/8 (reduction in PDAI ≥3 at 4 wk)	--	--	11.5 range 10–14	10.5 range 9–14	Total—7/8 [3/8 (nausea), 1/8 (vomiting), 2/8 (bloating), 1/8 (fever)]	None	NR
Stallmach 2016^23^	3/5 (reduction in PDAI ≥3 and total of <7 after last FMT)	1/5	4/5 (reduction in PDAI ≥3 at 4 wk)	--	--	9–14	2–7	Total—1/5 (mild transient fever)	None	3m
Stuebe 2017^22^	--	--	7/14 (clinical response)	--	--	--	--	--	None	--
Herfarth 2019^20^	--	4/5	0/4	1/5 (mPDAI <4 and no need for antibiotics in weeks 4, 8, and 16)	--	--	--	Total—0/4	None	4m (16 wk)
Nishida 2019^24^	0/3 (PDAI <7 at 8 wk)	1/3	1/3 (reduction in PDAI >3 points after 8 wk)	--	--	9–15	7–14	Total—0/3	None	2m (8 wk)
Kousgaard 2020^8^	0/9 (PDAI <7 at 4 wk)	6/9	4/9 (improvement in cPDAI at 4 wk)	3/9 (sustained improvement in cPDAI at 24 wk)	0/9 (at 4 wk)	8.6 (3.4)	5.2 (4.5)	Total 9/9 [5/9 (abdominal pain), 2/9 (nausea), 2/9 (fever)]	None	6m
Selvig 2020^2^	1/11 (reduction in PDAI ≥ 3 and total of <7 after 4 wk)	--	9/18 (subjective improvement at week 4)	--	4/11 (at wk 4)	7 (6–8)	6 (5.5–7.5)	Total—16/18 [4n (Abdominal pain or discomfort), 4 n (flatulence), 3 n (bloating or cramping), 3 n (fatigue), 2 n (nausea)]	One patient reported a norovirus infection 6 mo after receiving FMT, considered unrelated to FMT given the time frame	1m (4 wk)
Karjalainen 2021^25^	4/13 (PDAI <7 at 52 wk)	9/13	3/13 (subjective improvement at 4 wk)	0/13 (subjective improvement at 52 wk)	--	5.6 (2.7)	4.8 (2.7)	Total 4/13 [3 n (fever, abdominal pain, and fecal urgency), 1 n (nausea)]	None	12m (52 wk)

cPDAI, clinical pouchitis diseaseactivity index; NR, not reported.

### Meta-Analysis with Weighted Outcomes

A. Overall Remission: In patients with chronic pouchitis that received FMT, pooled proportion of patients with overall remission was 21.6% (95% CI, 9.2–43; I^2^, 28.5%). Figure 1.
B. Clinical Remission: In patients with chronic pouchitis that received FMT, pooled proportion of patients achieving clinical remission was 20.1% (95% CI, 6.2–48.7; I^2^, 24.6%). Figure 2.
C. Clinical Response: In patients with chronic pouchitis that received FMT, pooled proportion of patients with clinical response was 41.5% (95% CI, 30–54.4; I^2^, 7.4%). Figure 3.
D. Relapse: In patients with chronic pouchitis that received FMT, pooled proportion of patients with relapse was 60% (95% CI, 40%–77.4%, I^2^, 15.7%). Figure 4.
E. Adverse Events: Pooled proportion of patients with adverse events was 54.3% (95% CI, 21.3%–84%; I^2^, 72.7%) in FMT cohort. Regarding adverse events, 12 patients reported abdominal pain or discomfort, 8 patients reported nausea, 7 patients reported fever, 5 patients reported bloating or cramping, 4 patients reported flatulence, 3 patients reported fatigue, 1 patient reported vomiting, and 1 patient reported fecal urgency. None of the patients reported infection after FMT, apart from a patient who reported norovirus infection 6 months after FMT, which was thought to be unrelated given the time frame.^2^ Figure A3.

## Validation of Meta-Analysis Results

### Sensitivity Analysis

To assess whether any one study had a dominant effect on the meta-analysis, we excluded one study at a time and analyzed its effect on overall remission. No significant difference in the pooled proportions was noted with the exclusion of any study, Figure A4.

### Heterogeneity

We assessed dispersion of the calculated rates using the CI and I^2^ percentage values. The CI gives an idea of the range of the dispersion and I^2^ tells us what proportion of the dispersion is true vs chance.^26^ Overall, low heterogeneity was noted in the analysis for all outcomes except adverse events, where we noted had substantial heterogeneity. This is likely due to variation in definitions and types of adverse events that were reported by individual studies.

### Bias Assessment

Risk of bias was assessed for clinical remission among the 2 included RCTs. Overall, some concerns were raised about reported outcomes and the quality of evidence was rated as low. While Herfarth et al used Modified Pouchitis Disease Activity Index (mPDAI) <4 with corresponding mPDAI clinical subscores for each of the patient-reported outcomes of bowel frequency and urgency of ≤1 as their definition for clinical remission, patient reported subjective symptoms were used as outcome endpoints by Karjalainen et al. Publication bias was not assessed as the number of studies included in our analysis was less than 10.

### Patient, Intervention, Comparator and Outcome Analysis

**Figure 5:**
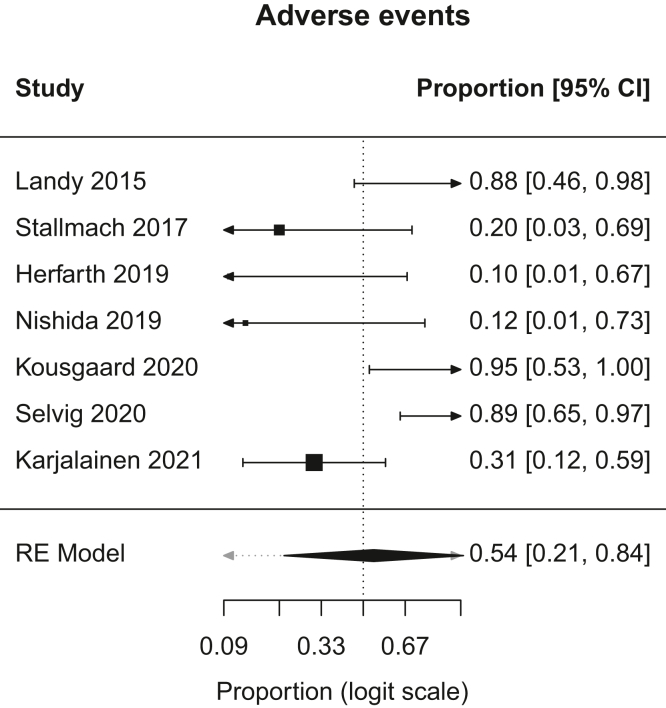
Forest plot of adverse events. RE, random effects.

1. Two studies included in this systematic review and meta-analysis compared FMT to no treatment in adults with chronic pouchitis (n = 30). Karjalainen et al (2021) found similar rates of relapse between the FMT (9/13) and placebo (8/13) groups. Herfarth et al (2019) found that out of 4 patients who received FMT, one achieved clinical remission as defined as mPDAI <4 with no need for antibiotics for the study period of 16 weeks following FMT. Due to the low sample sizes as described above, it is difficult to fully discern any differences in the effectiveness of FMT compared to no treatment in adult patients with chronic pouchitis in achieving response and remission.
2. None of the studies included in this systematic review and meta-analysis compared the effectiveness of FMT compared to antibiotics in achieving response and remission in adults with chronic pouchitis.
3. None of the studies included in this systematic review and meta-analysis compared the effectiveness of FMT compared to probiotics in achieving response and remission in adults with chronic pouchitis.
4. None of the studies included in this systematic review and meta-analysis compared the effectiveness of FMT after antibiotics compared to antibiotics alone in achieving response and remission in adults with chronic pouchitis.
5. None of the studies included in this systematic review and meta-analysis compared the effectiveness of FMT compared to biologics and small molecule therapies in achieving response and remission in adults with chronic pouchitis.
6. None of the studies included in this systematic review and meta-analysis assessed the effectiveness of FMT at preventing recurrent pouchitis.
7. Following FMT for chronic pouchitis, the most common adverse events were abdominal pain or discomfort, nausea, fever, flatulence, and fatigue. The pooled proportion of patients with adverse events was 54%. All of the reported adverse events were mild, and none led to cessation of therapy (Figure 5).

## Discussion

In this meta-analysis, 42% of patients with chronic pouchitis who received FMT experienced a clinical response at short term follow up. While over 50% of patients reported adverse events post FMT, these were largely minor and there were no reports of infection linked to FMT. FMT has potential as a therapeutic in chronic pouchitis, but larger studies are needed with more uniform protocols to elucidate the optimal route of administration and schedule of administration.

FMT has been demonstrated to be efficacious in treating recurrent *C. difficile* infection.^27^ FMT is hypothesized to be effective at treating pouchitis, as the gut microbiota mainly inhabit the distal ileum and pouchitis is thought to be a consequence of changes in bacterial composition of the terminal ileum secondary to surgical alterations in the bowel anatomy.^2^^,^^28^ Karjalainen et al (2021) conducted the largest study to date (n = 26), comparing FMT to placebo in patients with chronic pouchitis.^25^ While the FMT treatments were well-tolerated, there was no significant difference in relapse rate between the 2 groups. Herfarth et al (2019) performed a placebo-controlled double blind trial in patients with antibiotic-dependent pouchitis though encountered difficulty with FMT engraftment so clinical remission was observed in only 1 out of 5 patients treated with FMT.^20^ Another study of patients with chronic antibiotic-refractory pouchitis demonstrated 50% clinical response rate for patients receiving FMT, and a corresponding increase in microbiota diversity posttransplant.^22^

Biologic therapies have been explored as treatments for chronic pouchitis, revealing mixed results. A Study to Evaluate the Efficacy and Safety of Vedolizumab in the Treatment of Chronic Pouchitis trial, a randomized, double-blind, placebo-controlled study of vedolizumab as a treatment for chronic pouchitis (n = 102) assessed mPDAI remission at week 14 and week 34 after repeated dosing of vedolizumab (through week 30) and ciprofloxacin (through week 4). Vedolizumab was significantly more successful in inducing clinical remission (31.4% compared to 9.8% for placebo) at week 14 and sustained remission (27.5% compared to 5.9% placebo) at week 34.^29^ Kjaer et al investigated the use of adalimumab for 12 weeks compared to placebo as treatment for chronic pouchitis. Reduction in clinical PDAI ≥2 at any time during the study period was observed in 50% of patients in the adalimumab group and 43% of patients in the placebo group. However, there was no significant difference in patients achieving total PDAI remission at 12 weeks.^7^ A systematic review and meta-analysis on use of anti-tumor necrosis factor therapy in pouchitis (n = 313) revealed rates of short-term and long-term clinical remission of 50% and 52%, respectively.^30^ A larger systematic review and meta-analysis including 15 studies with 311 patients with chronic antibiotic refractory pouchitis, reported pooled rates of clinical improvement as 71.4%, 58.2%, 47.9% and those of clinical remission as 65.7%, 31%, 47.4% with anti-tumor necrosis factor therapy (infliximab and adalimumab) and vedolizumab, respectively.^31^ When comparing with the above rates of remission for biologic therapies, FMT may have similar efficacy in treatment of chronic pouchitis.

Generally, FMT is well tolerated, with overall incidence of adverse events reported as 28.5% in a systematic review.^32^ Incidence of adverse events was lower for FMT administered through the lower gastro intestinal tract. The most reported adverse event was abdominal discomfort, with flatulence, increased stool frequency, constipation, vomiting, and belching reported as well. Serious adverse events, such as viral/bacterial infections and death, occurred at a rate of 9.2%, though future research is warranted to reveal the degree to which these events were related to the FMT treatment.

In our analysis, we assessed the benefit of FMT through the maximum follow up included in each study. In several of the individual studies included in this systematic review, patients experienced improvement in subjective symptoms and had reduction in PDAI ≥3, meeting criteria for clinical response. Meanwhile, other studies reported limited clinical response with FMT. The greatest percentage of patients meeting criteria for clinical remission was 33.3%.^8^ Most importantly, the rate of relapse, when considering the studies in aggregate, was 55.7% in the cohort of patients treated with FMT. Notably, in A Study to Evaluate the Efficacy and Safety of Vedolizumab in the Treatment of Chronic Pouchitis trial discussed above remission rates were 31.8% for vedolizumab and 9.8% for placebo at week 14.^29^ A study of probiotic treatment of chronic pouchitis demonstrated a relapse rate of 15% in the probiotic group compared to 100% in the placebo group within the 9 month follow up period.^33^ Adverse events were relatively common, though most were mild, and none led to cessation of therapy. Considering the above results, based on a limited number of studies, it is difficult to make any definitive conclusions regarding the effectiveness of FMT in relieving pouchitis symptoms and contributing to complete remission.

There is significant variability in the subtype of chronic pouchitis of the included patients, use of antibiotics prior to FMT, and the dosage, frequency, and route of administration of FMT in the studies included in this systematic review and meta-analysis. Consequently, any trends regarding the most effective protocols for treatment of pouchitis with FMT may be obscured through this analysis. Studies on both antibiotic-refractory and antibiotic-dependent pouchitis are included and patients were included who had taken antibiotics from 2 weeks prior to FMT to 24 hours prior to FMT. All 8 studies involved a different route of administration of FMT, which ranged from upper endoscopy, to colonoscopy, pouchoscopy, transanal catheter, nasogastric tube, and enema. Dosage of FMT differed substantially as well, ranging from 18 g of fecal material to 200 g of fecal material. Further research is warranted to elucidate the optimal approach to using FMT for treatment of pouchitis.

There are several strengths to our review: we performed a systematic literature search with well-defined inclusion criteria, inclusion of good quality studies with detailed extraction of data, rigorous evaluation of study quality, and statistics to establish and/or refute the validity of the results of our meta-analysis. A previously published systematic review on the use of FMT in chronic pouchitis reported that clinical response after FMT was seen in 14 (31.8%) out of 44 evaluated patients at various timepoints after FMT, and clinical remission was reported in 10 (22.7%) patients.^34^ In our analysis, we excluded individual case reports and pooled our outcomes based on the uniformity of outcome definitions across the studies, including for overall remission as well as clinical response and remission. Furthermore, we evaluated clinical response at index follow up and remission at maximum follow-up to best assess the efficacy of FMT in chronic pouchitis patients. Despite this, we recognized that there was variation in definitions of clinical response and remission across the studies, either based on sustained improvement in clinical PDAI or subjective improvement in symptoms.

The mixed evidence supporting the use of FMT in pouchitis should also be interpreted in the context of the several limitations. First, all the included studies had small sample sizes with heterogeneous study designs, and only 2 RCTs were reviewed. Additionally, fewer studies included a placebo group for comparison. Second, we recognize the possibility of patient overlap in 2 of our included studies.^22^^,^^23^ While we attempted to contact the authors to clarify this, we did not receive a response. However, it is important to note that on our sensitivity analysis for overall remission, exclusion of no single study affected our meta-analysis outcomes. Third, there was significant heterogeneity in terms of FMT route of administration, dose of fecal material administered, and study follow up time further confounding the ability to draw clear conclusions from the results. Fourth, outcomes of endoscopic response were only reported by 2 studies. While, Kousgaard et al reported that none of the patients showed improvement by endoscopic PDAI score between inclusion and 30-day follow-up, including histologic PDAI scores, Selvig et al reported improvement in endoscopic PDAI in 4 out of 11 patients at 4 weeks.^2^^,^^8^ Overall, there was lack of agreement on what defines clinical response to FMT and what defines remission. Standardized outcome measures would help the analysis and interpretation of the true efficacy of FMT. Finally, the lack of high-quality Head-to-Head trials makes it difficult to measure the benefits of FMT.

Nevertheless, our study is the most comprehensive review evaluating the efficacy as well as safety of FMT in patients with pouchitis. At present, the treatment appears safe though there are mixed results in terms of FMT’s long term efficacy. It remains crucial that additional RCTs with larger sample sizes and greater standardization in terms of preparation, delivery, and length of treatment of FMT be performed in order to further elucidate our findings.
