# Supplementary material for: Safety and Efficacy of Fecal Microbiota Transplant in Chronic Pouchitis—A Systematic Review With Meta-Analysis
Source: Gastro Hep Adv. 2023 Apr 25;2(6):843–52. doi: 10.1016/j.gastha.2023.04.005 (PMC11307912; doi:10.1016/j.gastha.2023.04.005)

**Safety And Efficacy of Fecal Microbiota Transplant (FMT) In Chronic Pouchitis – A Systematic Review with Meta-analysis**

Tamara Kahan BA^1^, Saurabh Chandan MD^2^, Shahab R. Khan MBBS^3^, Smit Deliwala MD^4^, Shannon Chang MD, Jordan Axelrad MD MPH, Aasma Shaukat MD MPH ^1^

SUPPLEMENTARY MATERIAL:

APPENDIX-1: Literature search strategy

APPENDIX-2: MOOSE Checklist

APPENDIX-3: PRISMA Checklist

Supplementary Table 1A-B: NHLB Study Quality Assessment for Cohort Studies and Case Series

Supplementary Figure 1: PRISMA Flow Diagram

Supplementary Figure 2: Cochrane Collaboration tool to assess risk of bias

Supplementary Figure3: Forest plot, overall adverse events

Supplementary Figure 4: Sensitivity Analysis, Overall remission

**APPENDIX-1: Literature search strategy**

The literature search was modeled on: Cold, F., Kousgaard, S. J., Halkjaer, S. I., Petersen, A. M., Nielsen, H. L., Thorlacius-Ussing, O., & Hansen, L. H. (2020). Fecal Microbiota Transplantation in the Treatment of Chronic Pouchitis: A Systematic Review. *Microorganisms*, *8*(9), 1433. <https://doi.org/10.3390/microorganisms8091433>

Searches were conducted in PubMed, as well as in Medline, EMBASE, and Cochrane Central Register of Controlled Trials via the Ovid platform. Each search strategy included a combination of keywords and controlled vocabulary appropriate to each database. The search was conducted on March 29, 2022 and was not limited by language or publication date.

Complete Search Strategy:

PubMed: 3/29/2022

(((("Fecal Microbiota Transplantation"[Mesh]) OR fmt[tw]) OR (((fecal OR faecal OR bacteria* OR feces OR faeces OR stool OR intestinal OR microbiota OR microflora)) AND (transfer* OR transplant OR transplantations OR transplantation OR infusion* OR bacteriotherapy)))) AND (((("Pouchitis"[Mesh]) OR "Colonic Pouches"[Mesh]) OR "Proctocolectomy, Restorative"[Mesh]) OR ((pouch* OR IPAA OR "j pouch" OR "ileal pouch")))

517 results

**Medline (Ovid): 3/29/2022**

1 exp Fecal Microbiota Transplantation/ 2308

2 "fmt".mp. 2846

3 exp feces/ or exp intestines/ or exp microbiota/ or exp gastrointestinal microbiome/ or (fecal or faecal or bacteria* or feces or faeces or stool or intestinal or microbiota or microflora).mp. 2253436

4 exp transplantation/ or (transfer* or transplant or transplantations or transplantation or infusion* or bacteriotherapy).mp. 1889793

5 3 and 4 154143

6 1 or 2 or 5 155177

7 exp pouchitis/ 930

8 exp colonic pouches/ 1811

9 exp proctocolectomy, restorative/ 3586

10 (pouch* or IPAA or "j pouch" or "ileal pouch").mp. 23099

11 7 or 8 or 9 or 10 24027

12 6 and 11 447

447 results

**EMBASE: 3/29/2022**

1 exp Fecal Microbiota Transplantation/ 6523

2 "fmt".mp. 4638

3 exp feces/ or exp intestines/ or exp microbiota/ or exp gastrointestinal microbiome/ or (fecal or faecal or bacteria* or feces or faeces or stool or intestinal or microbiota or microflora).mp. 2248661

4 exp transplantation/ or (transfer* or transplant or transplantations or transplantation or infusion* or bacteriotherapy).mp. 2620325

5 3 and 4 184046

6 1 or 2 or 5 185604

7 exp pouchitis/ 6396

8 exp colonic pouches/ 1764

9 exp proctocolectomy, restorative/ 7032

10 (pouch* or IPAA or "j pouch" or "ileal pouch").mp. 32435

11 7 or 8 or 9 or 10 39757

12 6 and 11 938

938 results

**Cochrane: 3/29/2022**

1 exp Fecal Microbiota Transplantation/ 0

2 "fmt".mp. 628

3 exp feces/ or exp intestines/ or exp microbiota/ or exp gastrointestinal microbiome/ or (fecal or faecal or bacteria* or feces or faeces or stool or intestinal or microbiota or microflora).mp. 76901

4 exp transplantation/ or (transfer* or transplant or transplantations or transplantation or infusion* or bacteriotherapy).mp. 137852

5 3 and 4 6769

6 1 or 2 or 5 6837

7 exp pouchitis/ 41

8 exp colonic pouches/ 52

9 exp proctocolectomy, restorative/ 110

10 (pouch* or IPAA or "j pouch" or "ileal pouch").mp. 1127

11 7 or 8 or 9 or 10 1148

12 6 and 11 33

33 results

**APPENDIX-2: MOOSE Checklist**

| Item No | Recommendation | Reported on Page No |
| --- | --- | --- |
| Reporting of background should include | | |
| 1 | Problem definition | 4-5 |
| 2 | Hypothesis statement | 5 |
| 3 | Description of study outcome(s) | 5 |
| 4 | Type of exposure or intervention used | 5 |
| 5 | Type of study designs used | 5 |
| 6 | Study population | 6 |
| Reporting of search strategy should include | | |
| 7 | Qualifications of searchers (eg, librarians and investigators) | 5 |
| 8 | Search strategy, including time period included in the synthesis and key words | 5 |
| 9 | Effort to include all available studies, including contact with authors | 6 |
| 10 | Databases and registries searched | 5 |
| 11 | Search software used, name and version, including special features used (eg, explosion) | 5 |
| 12 | Use of hand searching (eg, reference lists of obtained articles) | -NA- |
| 13 | List of citations located and those excluded, including justification | 8-9,  Suppl Fig 1 |
| 14 | Method of addressing articles published in languages other than English | -NA- |
| 15 | Method of handling abstracts and unpublished studies | 6 |
| 16 | Description of any contact with authors | -NA- |
| Reporting of methods should include | | |
| 17 | Description of relevance or appropriateness of studies assembled for assessing the hypothesis to be tested | 5-6 |
| 18 | Rationale for the selection and coding of data (eg, sound clinical principles or convenience) | 6 |
| 19 | Documentation of how data were classified and coded (eg, multiple raters, blinding and interrater reliability) | 6 |
| 20 | Assessment of confounding (eg, comparability of cases and controls in studies where appropriate) | 6 |
| 21 | Assessment of study quality, including blinding of quality assessors, stratification or regression on possible predictors of study results | 7 |
| 22 | Assessment of heterogeneity | 8 |
| 23 | Description of statistical methods (eg, complete description of fixed or random effects models, justification of whether the chosen models account for predictors of study results, dose-response models, or cumulative meta-analysis) in sufficient detail to be replicated | 8 |
| 24 | Provision of appropriate tables and graphics | Tables 1-2, Figs 1-4, supple fig 3.4 |
| Reporting of results should include | | |
| 25 | Graphic summarizing individual study estimates and overall estimate | Fig 1-4 |
| 26 | Table giving descriptive information for each study included | Table 1-2 |
| 27 | Results of sensitivity testing (eg, subgroup analysis) | 12-13 |
| 28 | Indication of statistical uncertainty of findings | 12-13 |

**APPENDIX-3: PRISMA CHECK List**

| **Section/Topic** | **Item #** | **Checklist Item** | **Reported on Page #** |
| --- | --- | --- | --- |
| **TITLE** |  |  |  |
| Title | 1 | Identify the report as a systematic review *incorporating a meta-analysis.* | **1** |
| **ABSTRACT** |  |  |  |
| Structured summary | 2 | Provide a structured summary including, as applicable:  **Background:** main objectives  **Methods:** data sources; study eligibility criteria, participants, and interventions; study appraisal; and synthesis methods  **Results:** number of studies and participants identified; summary estimates with corresponding confidence/credible intervals and implications of findings.  **Conclusion:** Implications of finds. | **3** |
|  |  |  |  |
| **INTRODUCTION** |  |  |  |
| Rationale | 3 | Describe the rationale for the review in the context of what is already known*, including mention of artificial intelligence (AI) and why a meta-analysis has been conducted.* | **4-5** |
| Objectives | 4 | Provide an explicit statement of questions being addressed, with reference to participants, interventions, comparisons, outcomes, and study design (PICOS). | **5** |
|  |  |  |  |
| **METHODS** |  |  |  |
| Protocol and registration | 5 | Indicate whether a review protocol exists and if and where it can be accessed (e.g., Web address); and, if available, provide registration information, including registration number. | **5** |
| Eligibility criteria | 6 | Specify study characteristics (e.g., PICOS, length of follow-up) and report characteristics (e.g., years considered, language, publication status) used as criteria for eligibility, giving rationale. | **6** |
| Information sources | 7 | Describe all information sources (e.g., databases with dates of coverage, contact with study authors to identify additional studies) in the search and date last searched. | **6** |
| Search | 8 | Present full electronic search strategy for at least one database, including any limits used, such that it could be repeated. | **6** |
| Study selection | 9 | State the process for selecting studies (i.e., screening, eligibility, included in systematic review, and, if applicable, included in the meta-analysis). | **6** |
| Data collection process | 10 | Describe method of data extraction from reports (e.g., piloted forms, independently, in duplicate) and any processes for obtaining and confirming data from investigators. | **6** |
| Data items | 11 | List and define all variables for which data were sought (e.g., PICOS, funding sources) and any assumptions and simplifications made. | **7** |
| Risk of bias within individual studies | 12 | Describe methods used for assessing risk of bias of individual studies (including specification of whether this was done at the study or outcome level), and how this information is to be used in any data synthesis. | **7** |
| Summary measures | 13 | State the principal summary measures (e.g., risk ratio, difference in means). | **7** |
| Planned methods of analysis | 14 | Describe the methods of handling data and combining results of studies for each network meta-analysis. | **7** |
| Risk of bias across studies | 15 | Specify any assessment of risk of bias that may affect the cumulative evidence (e.g., publication bias, selective reporting within studies). | **7** |
| Additional analyses | 16 | Describe methods of additional analyses if done, indicating which were pre-specified. This may include, but not be limited to, the following:   - Sensitivity analysis - Subgroup analysis | **7** |
| **RESULTS** |  |  |  |
| Study selection | 17 | Give numbers of studies screened, assessed for eligibility, and included in the review, with reasons for exclusions at each stage, ideally with a flow diagram. | **7** |
| Study characteristics | 18 | For each study, present characteristics for which data were extracted (e.g., study size, PICOS, follow-up period) and provide the citations. | **8** |
| Risk of bias within studies | 19 | Present data on risk of bias of each study and, if available, any outcome level assessment (see item 12). | **9** |
| Results of individual studies | 20 | For all outcomes considered (benefits or harms), present, for each study: 1) simple summary data for each intervention group, and 2) effect estimates and confidence intervals using a forest plot. | **10** |
| Synthesis of results | 21 | Present results of each meta-analysis done, including confidence/credible intervals. If additional summary measures were explored (such as treatment rankings), these should also be presented. | **11-12** |
| Results of additional analyses | 23 | Give results of additional analyses, if done   - Sensitivity analysis - Subgroup analysis | **11-12** |
| Risk of bias across studies | 22 | Present results of any assessment of risk of bias across studies for the evidence base being studied (see item 15). | **13** |
| **DISCUSSION** |  |  |  |
| Summary of evidence | 24 | Summarize the main findings, including the strength of evidence for each main outcome; consider their relevance to key groups (e.g., healthcare providers, users, and policy-makers). | **13-15** |
| Limitations | 25 | Discuss limitations at study and outcome level (e.g., risk of bias), and at review level (e.g., incomplete retrieval of identified research, reporting bias). | **15** |
| Conclusions | 26 | Provide a general interpretation of the results in the context of other evidence, and implications for future research. | **17** |
| **FUNDING** |  |  |  |
| Funding | 27 | Describe sources of funding for the systematic review and other support (e.g., supply of data); role of funders for the systematic review. This should also include information regarding whether funding has been received from manufacturers of treatments in the network and/or whether some of the authors are content experts with professional conflicts of interest that could affect use of treatments in the network. | **Disclosed in title page** |

Supplementary Table 1A: Study Quality Assessment – Cohort Studies

| CRITERIA | Was the research question or objective in this paper clearly stated? | Was the study population clearly specified and defined? | Was the participation rate of eligible persons at least 50%? | Were all the subjects selected or recruited from the same or similar populations (including the same time period)? Were inclusion and exclusion criteria for being in the study prespecified and applied uniformly to all participants? | Was a sample size justification, power description, or variance and effect estimates provided? | For the analyses in this paper, were the exposure(s) of interest measured prior to the outcome(s) being measured? | Was the timeframe sufficient so that one could reasonably expect to see an association between exposure and outcome if it existed? | For exposures that can vary in amount or level, did the study examine different levels of the exposure as related to the outcome (e.g., categories of exposure, or exposure measured as continuous variable)? | Were the exposure measures (independent variables) clearly defined, valid, reliable, and implemented consistently across all study participants? | Was the exposure(s) assessed more than once over time? | Were the outcome measures (dependent variables) clearly defined, valid, reliable, and implemented consistently across all study participants? | Were the outcome assessors blinded to the exposure status of participants? | Was loss to follow-up after baseline 20% or less? | Were key potential confounding variables measured and adjusted statistically for their impact on the relationship between exposure(s) and outcome(s)? | QUALITY |
| --- | --- | --- | --- | --- | --- | --- | --- | --- | --- | --- | --- | --- | --- | --- | --- |
| Stuebe 2017 | Y | Y | Y | Y | N | N | Y | Y | Y | NA | Y | NA | NA | NA | POOR |
| Kousgaard 2020 | Y | Y | Y | Y | Y | N | Y | Y | Y | Y | Y | N | Y | N | GOOD |
| Selvig 2020 | Y | Y | Y | Y | Y | N | Y | Y | Y | Y | Y | N | Y | N | GOOD |

Supplementary Table 1B: Study Quality Assessment – Case Series

| CRITERIA | Was the research question or objective in this paper clearly stated? | Was the study population clearly and fully described, including a case definition? | Were the cases consecutive? | Were the subjects comparable? | Was the intervention clearly described? | Were the outcome measures clearly defined, valid, reliable, and implemented consistently across all study participants? | Was the length of follow-up adequate? | Were the statistical methods well-described? | Were the results well-described? | Quality |
| --- | --- | --- | --- | --- | --- | --- | --- | --- | --- | --- |
| Landy 2015 | Y | Y | Y | Y | Y | Y | NA | Y | Y | GOOD |
| Stallmach 2017 | Y | Y | Y | Y | Y | Y | Y | Y | Y | GOOD |
| Nishida 2019 | Y | Y | Y | Y | Y | Y | Y | Y | Y | GOOD |

Supplementary Figure 1 – PRISMA Flow Chart

Records screened
(n = 1450)

Records after duplicates removed
(n = 1450)

## Identification

## Eligibility

## Included

## Screening

Records identified through database searching
(n = 1935)

Records excluded based on abstract/title
(n = 1252)

Full-text articles excluded, with reasons
(n = 190)

(Case reports, review articles, non pouchitis studies)

S

Full-text articles assessed for eligibility
(n = 198)

Studies included in quantitative synthesis (meta-analysis)
(n = 8)

Supplementary Figure 2 - Risk of bias assessment

## Risk of bias assessment - Karjalainen 2021 – Clinical Remission

Responses underlined in green are potential markers for low risk of bias, and responses in red are potential markers for a risk of bias. Where questions relate only to sign posts to other questions, no formatting is used.

**Domain 1: Risk of bias arising from the randomization process**

| **Signalling questions** | **Comments** | **Response options** |
| --- | --- | --- |
| **1.1 Was the allocation sequence random?** |  | Y |
| **1.2 Was the allocation sequence concealed until participants were enrolled and assigned to interventions?** |  | Y |
| **1.3 Did baseline differences between intervention groups suggest a problem with the randomization process?** |  | N |
| **Risk-of-bias judgement** |  | Low |
| Optional: What is the predicted direction of bias arising from the randomization process? |  | NA |

Domain 2: Risk of bias due to deviations from the intended interventions (*effect of assignment to intervention*)

| **Signalling questions** | **Comments** | **Response options** |
| --- | --- | --- |
| **2.1. Were participants aware of their assigned intervention during the trial?** |  | N |
| **2.2. Were carers and people delivering the interventions aware of participants' assigned intervention during the trial?** |  | N |
| **2.3. If Y/PY/NI to 2.1 or 2.2: Were there deviations from the intended intervention that arose because of the trial context?** |  | NA |
| **2.4 If Y/PY to 2.3: Were these deviations likely to have affected the outcome?** |  | NA |
| **2.5. If Y/PY/NI to 2.4: Were these deviations from intended intervention balanced between groups?** |  | NA |
| **2.6 Was an appropriate analysis used to estimate the effect of assignment to intervention?** |  | Y |
| **2.7 If N/PN/NI to 2.6: Was there potential for a substantial impact (on the result) of the failure to analyse participants in the group to which they were randomized?** |  | NA |
| **Risk-of-bias judgement** |  | Low |
| Optional: What is the predicted direction of bias due to deviations from intended interventions? |  | NA |

Domain 2: Risk of bias due to deviations from the intended interventions (*effect of adhering to intervention*)

| **Signalling questions** | **Comments** | **Response options** |
| --- | --- | --- |
| **2.1. Were participants aware of their assigned intervention during the trial?** |  | N |
| **2.2. Were carers and people delivering the interventions aware of participants' assigned intervention during the trial?** |  | N |
| **2.3. [If applicable:] If Y/PY/NI to 2.1 or 2.2: Were important non-protocol interventions balanced across intervention groups?** |  | NA |
| **2.4. [If applicable:] Were there failures in implementing the intervention that could have affected the outcome?** |  | NA |
| **2.5. [If applicable:] Was there non-adherence to the assigned intervention regimen that could have affected participants’ outcomes?** |  | NA |
| **2.6. If N/PN/NI to 2.3, or Y/PY/NI to 2.4 or 2.5: Was an appropriate analysis used to estimate the effect of adhering to the intervention?** |  | NA |
| **Risk-of-bias judgement** |  | Low |
| Optional: What is the predicted direction of bias due to deviations from intended interventions? |  | NA |

Domain 3: Missing outcome data

| **Signalling questions** | **Comments** | **Response options** |
| --- | --- | --- |
| **3.1 Were data for this outcome available for all, or nearly all, participants randomized?** |  | Y |
| **3.2 If N/PN/NI to 3.1: Is there evidence that the result was not biased by missing outcome data?** |  | NA |
| **3.3 If N/PN to 3.2: Could missingness in the outcome depend on its true value?** |  | NA |
| **3.4 If Y/PY/NI to 3.3: Is it likely that missingness in the outcome depended on its true value?** |  |  |
| **Risk-of-bias judgement** |  | Low |
| Optional: What is the predicted direction of bias due to missing outcome data? |  | NA |

Domain 4: Risk of bias in measurement of the outcome

| **Signalling questions** | **Comments** | **Response options** |
| --- | --- | --- |
| **4.1 Was the method of measuring the outcome inappropriate?** |  | N |
| **4.2 Could measurement or ascertainment of the outcome have differed between intervention groups?** |  | PY |
| **4.3 If N/PN/NI to 4.1 and 4.2: Were outcome assessors aware of the intervention received by study participants?** |  | NA |
| **4.4 If Y/PY/NI to 4.3: Could assessment of the outcome have been influenced by knowledge of intervention received?** |  | NA |
| **4.5 If Y/PY/NI to 4.4:** **Is it likely that assessment of the outcome was influenced by knowledge of intervention received?** |  | NA |
| **Risk-of-bias judgement** |  | Some concerns |
| Optional: What is the predicted direction of bias in measurement of the outcome? |  | NA |

Domain 5: Risk of bias in selection of the reported result

| **Signalling questions** | **Comments** | **Response options** |
| --- | --- | --- |
| **5.1 Were the data that produced this result analysed in accordance with a pre-specified analysis plan that was finalized before unblinded outcome data were available for analysis?** |  | Y |
| **Is the numerical result being assessed likely to have been selected, on the basis of the results, from...** |  |  |
| **5.2. ... multiple eligible outcome measurements (e.g. scales, definitions, time points) within the outcome domain?** |  | Y |
| **5.3 ... multiple eligible analyses of the data?** |  | PY |
| **Risk-of-bias judgement** |  | Low |
| Optional: What is the predicted direction of bias due to selection of the reported result? |  | NA |

Overall risk of bias

| **Risk-of-bias judgement** |  | Some concerns |
| --- | --- | --- |
| Optional: What is the overall predicted direction of bias for this outcome? |  | NA |

## Risk of bias assessment - Herfarth 2019 – Clinical Remission

Responses underlined in green are potential markers for low risk of bias, and responses in red are potential markers for a risk of bias. Where questions relate only to sign posts to other questions, no formatting is used.

**Domain 1: Risk of bias arising from the randomization process**

| **Signalling questions** | **Comments** | **Response options** |
| --- | --- | --- |
| **1.1 Was the allocation sequence random?** |  | Y |
| **1.2 Was the allocation sequence concealed until participants were enrolled and assigned to interventions?** |  | Y |
| **1.3 Did baseline differences between intervention groups suggest a problem with the randomization process?** |  | N |
| **Risk-of-bias judgement** |  | Low |
| Optional: What is the predicted direction of bias arising from the randomization process? |  | NA |

Domain 2: Risk of bias due to deviations from the intended interventions (*effect of assignment to intervention*)

| **Signalling questions** | **Comments** | **Response options** |
| --- | --- | --- |
| **2.1. Were participants aware of their assigned intervention during the trial?** |  | N |
| **2.2. Were carers and people delivering the interventions aware of participants' assigned intervention during the trial?** |  | N |
| **2.3. If Y/PY/NI to 2.1 or 2.2: Were there deviations from the intended intervention that arose because of the trial context?** |  | NA |
| **2.4 If Y/PY to 2.3: Were these deviations likely to have affected the outcome?** |  | NA |
| **2.5. If Y/PY/NI to 2.4: Were these deviations from intended intervention balanced between groups?** |  | NA |
| **2.6 Was an appropriate analysis used to estimate the effect of assignment to intervention?** |  | Y |
| **2.7 If N/PN/NI to 2.6: Was there potential for a substantial impact (on the result) of the failure to analyse participants in the group to which they were randomized?** |  | NA |
| **Risk-of-bias judgement** |  | Low |
| Optional: What is the predicted direction of bias due to deviations from intended interventions? |  | NA |

Domain 2: Risk of bias due to deviations from the intended interventions (*effect of adhering to intervention*)

| **Signalling questions** | **Comments** | **Response options** |
| --- | --- | --- |
| **2.1. Were participants aware of their assigned intervention during the trial?** |  | N |
| **2.2. Were carers and people delivering the interventions aware of participants' assigned intervention during the trial?** |  | N |
| **2.3. [If applicable:] If Y/PY/NI to 2.1 or 2.2: Were important non-protocol interventions balanced across intervention groups?** |  | NA |
| **2.4. [If applicable:] Were there failures in implementing the intervention that could have affected the outcome?** |  | NA |
| **2.5. [If applicable:] Was there non-adherence to the assigned intervention regimen that could have affected participants’ outcomes?** |  | NA |
| **2.6. If N/PN/NI to 2.3, or Y/PY/NI to 2.4 or 2.5: Was an appropriate analysis used to estimate the effect of adhering to the intervention?** |  | NA |
| **Risk-of-bias judgement** |  | Low |
| Optional: What is the predicted direction of bias due to deviations from intended interventions? |  | NA |

Domain 3: Missing outcome data

| **Signalling questions** | **Comments** | **Response options** |
| --- | --- | --- |
| **3.1 Were data for this outcome available for all, or nearly all, participants randomized?** |  | Y |
| **3.2 If N/PN/NI to 3.1: Is there evidence that the result was not biased by missing outcome data?** |  | NA |
| **3.3 If N/PN to 3.2: Could missingness in the outcome depend on its true value?** |  | NA |
| **3.4 If Y/PY/NI to 3.3: Is it likely that missingness in the outcome depended on its true value?** |  | NA |
| **Risk-of-bias judgement** |  | Low |
| Optional: What is the predicted direction of bias due to missing outcome data? |  | NA |

Domain 4: Risk of bias in measurement of the outcome

| **Signalling questions** | **Comments** | **Response options** |
| --- | --- | --- |
| **4.1 Was the method of measuring the outcome inappropriate?** |  | N |
| **4.2 Could measurement or ascertainment of the outcome have differed between intervention groups?** |  | PY |
| **4.3 If N/PN/NI to 4.1 and 4.2: Were outcome assessors aware of the intervention received by study participants?** |  | NA |
| **4.4 If Y/PY/NI to 4.3: Could assessment of the outcome have been influenced by knowledge of intervention received?** |  | NA |
| **4.5 If Y/PY/NI to 4.4: Is it likely that assessment of the outcome was influenced by knowledge of intervention received?** |  | NA |
| **Risk-of-bias judgement** |  | Some concerns |
| Optional: What is the predicted direction of bias in measurement of the outcome? |  | NA |

Domain 5: Risk of bias in selection of the reported result

| **Signalling questions** | **Comments** | **Response options** |
| --- | --- | --- |
| **5.1 Were the data that produced this result analysed in accordance with a pre-specified analysis plan that was finalized before unblinded outcome data were available for analysis?** |  | Y |
| **Is the numerical result being assessed likely to have been selected, on the basis of the results, from...** |  |  |
| **5.2. ... multiple eligible outcome measurements (e.g. scales, definitions, time points) within the outcome domain?** |  | PY |
| **5.3 ... multiple eligible analyses of the data?** |  | PY |
| **Risk-of-bias judgement** |  | Some concerns |
| Optional: What is the predicted direction of bias due to selection of the reported result? |  | NA |

Overall risk of bias

| **Risk-of-bias judgement** |  | Some concerns |
| --- | --- | --- |
| Optional: What is the overall predicted direction of bias for this outcome? |  | NA |

Supplementary Figure3: Forest plot, overall adverse events


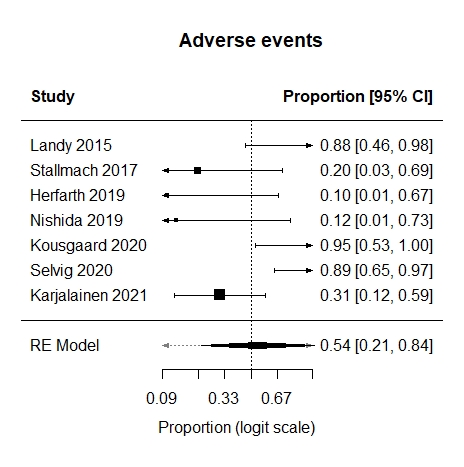


Supplementary Figure 4: Sensitivity Analysis, Overall remission


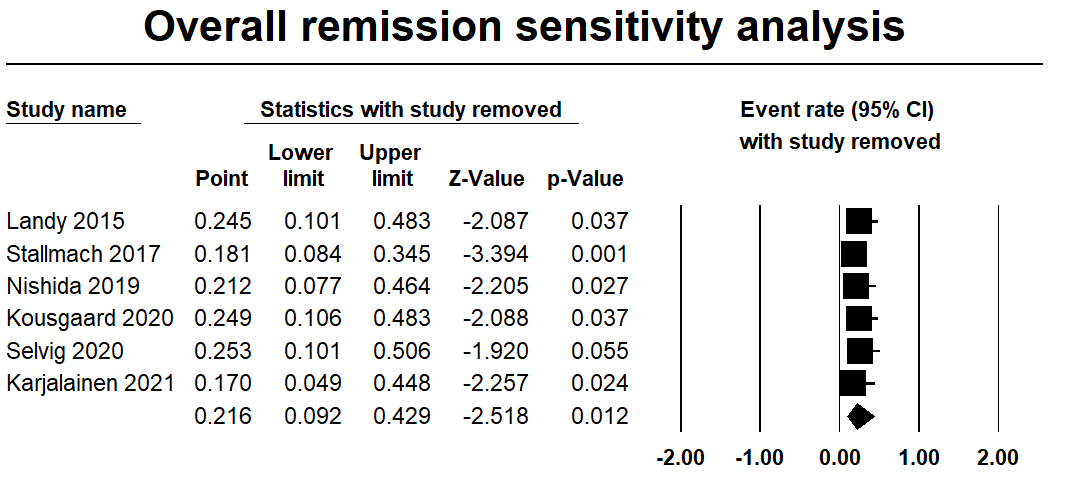

Supplement: Supplementary Figures S1–S3 and Tables S1–S4 [file mmc1.docx]
